# Dehydration affects drug transport over nasal mucosa

**DOI:** 10.1080/10717544.2019.1650848

**Published:** 2019-08-10

**Authors:** Abdullah Ali, Marie Wahlgren, Birgitta Rembratt-Svensson, Ameena Daftani, Peter Falkman, Per Wollmer, Johan Engblom

**Affiliations:** a Biomedical Sciences, Faculty of Health and Society, Malmö University, Malmö, Sweden;; b Biofilms – Research Center for Biointerfaces, Malmö University, Malmö, Sweden;; c Food Technology, Engineering and Nutrition, Lund University, Lund, Sweden;; d Bioglan AB, Malmö, Sweden;; e Department of Translational Medicine, Faculty of Medicine, Lund University, Malmö, Sweden

**Keywords:** Mucoadhesion, nasal drug delivery, dehydration, water activity, drug transport

## Abstract

Formulations for nasal drug delivery often rely on water sorption to adhere to the mucosa, which also causes a higher water gradient over the tissue and subsequent dehydration. The primary aim of this study was therefore to evaluate mucosal response to dehydration and resolve the hypothesis that mucoadhesion achieved through water sorption could also be a constraint for drug absorption via the nasal route. The effect of altering water activity of the vehicle on Xylometazoline HCl and ^51^Cr-EDTA uptake was studied separately *ex vivo* using flow through diffusion cells and excised porcine mucosa. We have shown that a modest increase in the water gradient over mucosa induces a substantial decrease in drug uptake for both Xylometazoline HCl and ^51^Cr-EDTA. A similar result was obtained when comparing two different vehicles on the market; Nasoferm^®^ (Nordic Drugs, Sweden) and BLOX4^®^ (Bioglan, Sweden). Mucoadhesion based on water sorption can slow down drug uptake in the nasal cavity. However, a clinical study is required to determine whether prolonged duration of the vehicle *in situ* or preventing dehydration of the mucosa is the most important factor for improving bioavailability.

## Introduction

The nasal route of drug delivery offers many advantages such as high absorption, avoidance of the first pass metabolism, rapid onset of effect and the possibility to circumvent the blood–brain barrier. In addition, due to the large surface area of the nose the nasal cavity offers good absorption for low molecular weight lipophilic drugs with bioavailability close to that of the intravenous route (Davis & Illum, 2003). One of the key advantages are that nasal delivery avoids parenteral injections which contributes to good patient-compliance while still in most cases providing higher bioavailability than oral administration (Wadell, 2002; Davis & Illum, 2003; Kumar et al., 2016). Moreover, nasal administration can be used both for local and for systemic drug delivery. Currently, there are numerous drug formulations used for nasal drug delivery for different indications such as analgesia, acute migraine, nasal congestions and infections (Davis & Illum, 2003; Illum, 2003; Ghori et al., 2015).

Some of the limitations encountered with nasal drug delivery is low permeability of polar molecules and large molecular weight peptides and proteins, as well as the mucociliary clearance (MCC). MCC limits transmucosal absorption by renewing the mucus layer lining of the mucosa every 15-21 minutes (Soane et al., 1999; Davis & Illum, 2003; Illum, 2003). The mucus layer can also limit absorption by binding the drug to mucin, the principle protein in the mucus. Smaller particles pass easily, while larger or charged particles could get trapped in the gel (Jadhav et al., 2007). After passing the mucus layer, the principle mechanisms of drug absorption through the mucosa include transcellular passive diffusion, paracellular passive diffusion and transcytosis by vesicle carriers (Wadell, 2002; Ugwoke et al., 2005; Jadhav et al., 2007).

Nasal formulations are usually used as solutions, gels, or powders. Other formulation types include suspensions, emulsions and microparticle formulations. Solutions are simple and convenient with good patient-compliance and easy administration through, for example, spray pumps (Lee et al., 2000; Upadhyay et al., 2011). They do however often suffer from poor retention characteristics preventing prolonged close contact with the absorption site. One approach to circumvent MCC is to use mucoadhesive polymer-based gels instead, for example self-gelling systems. Gels are highly desirable when comprising bioadhesive polymers that can prolong contact time at the absorption site and improve bioavailability (Smart, 2005; Ugwoke et al., 2005; Duan & Mao, 2010). Powders are less frequently used but have advantages of prolonged contact time with mucosa due to, for example, water sorption and the possibility to formulate preservative-free products. Polymer and powder-based formulations often rely on water sorption and swelling to adhere to the mucosa. Moreover, aqueous polymer formulations often exhibit decrease in water activity (Ninni et al., 1999; Björklund et al., 2010), which induces a higher water gradient over the tissue and subsequent dehydration (Mortazavi & Smart, 1993; Pereswetoff-Morath & Morath, 1998; Marshall et al., 2001, 2004). Several studies have indeed reported an increase in nasal drug delivery following use of bioadhesive formulations to prolong the contact time (Björk & Edman, 1990; Pereswetoff-Morath & Morath, 1998; Ugwoke et al., 2005), while others report on lower permeability (Hansen et al., 2015) and build-up of a physical barrier after repeated administration (Callens et al., 2003). Some studies have furthermore focused on studying barrier forming formulations preventing, for example, allergenic rhinitis (Josling & Steadman, 2003; Schwetz et al., 2004; Emberlin & Lewis, 2006; Andersson et al., 2008, 2014). However, none of these studies has discussed the transport-barrier response of the mucosa affecting drug permeability, caused by changes in the water gradient across the mucosa subsequent to formulation administration. When a nasal formulation is administered into the nasal cavity, a water gradient across the nasal mucosa will be induced by difference in water chemical potential of the formulation on the outer part of the mucosa, and the inner side of the mucosa where it is constant (physiological conditions). Previous research has shown that an increased water gradient can be detrimental to drug absorption over both skin and oral mucosa (Björklund et al., 2010; Albèr et al., 2013; Ali et al., 2018). Recent studies on pig gastric mucin have also shown how the mobility of small molecules decrease when the water activity in mucin gels is decreased (Runnsjö et al., 2016). It is not farfetched that application of polymer-based formulations to the nasal mucosa may induce a similar response detrimental to drug absorption.

The primary aim of this study was to evaluate mucosal response to changes in water gradient and resolve the hypothesis that mucoadhesion achieved through water sorption could also be a constraint for drug absorption via the nasal route. In other words, we are interested in how the water activity of formulations affects the nasal mucosa and its permeability.

We have investigated the permeability of two hydrophilic substances, Xylometazoline HCl and radiolabeled Chromium-51 ethylene diamine tetraacetate (^51^Cr-EDTA), *ex vivo* across porcine nasal mucosa in aqueous solutions where the water activity of the vehicle has been controlled using polyethylene glycol 1500 (PEG1500) (Ninni et al., 1999; Björklund et al., 2010). Xylometazoline HCl is a commonly used nasal decongestant, which when administered to nasal mucosa leads to reduction of mucus and liquid production. It is suitable for treating colds, irritation and congestion of the nasal mucosa induced by allergies. ^51^Cr-EDTA has been identified as an appropriate model drug for studying absorption through nasal epithelium *in vivo*. It has been used in clinical studies focused on the physiology of the nose (Andersson et al., 2011). It is a safe and stable hydrophilic molecule with a similar size (*M*_W_ = 339 g/mol) to Xylometazoline HCl (*M*_W_ = 281 g/mol) and with high recovery in urine (>90%) (Downes & McDonald, 1964; Greiff et al., 1991). ^51^Cr-EDTA is included here with a future clinical study in mind and the possibility to provide a platform for evaluating *ex vivo-in vivo* correlation (EVIVC). A clinical study is expected to provide answers related to duration of the formulation in the nasal cavity, drug absorption through nasal mucosa and the effect of changing the gradient in water activity over the mucosa. The advantages of *ex vivo* studies over *in vivo* studies are that they are faster, fewer animals are required, and by avoiding the presence of plasma proteins in the samples more simple analytical procedures can be used (Lee et al., 1997). *In vivo* studies are nevertheless essential for validation of *ex vivo* results. Clinical studies usually use radiolabeled markers and focus mainly on the physiology of the nasal epithelium (Greiff et al., 1993, 1994; Andersson et al., 2008). A clinical study allows testing the drug formulation on human mucosa with pre- and postmucosal factors such as the active MCC, having a cold, and any enzymatic degradation that can take place. Furthermore, as chelating agents have been reported to act as absorption enhancers (Davis & Illum, 2003; Jadhav et al., 2007), it was decided to investigate if addition of Na_2_-EDTA (pKa = 2.0, 2.7, 6.2, 10.3, *M*_W_ = 372 g/mol) (Dawson, 1986) affects absorption of ^51^Cr-EDTA.

Two commercial products, Nasoferm^®^ (Nordic Drugs AB, Sweden) and BLOX4^®^ (Bioglan AB, Sweden), were appended to compare different types of vehicle systems with respect to Xylometazoline HCl permeation over mucosa, *ex vivo*. Nasoferm^®^ is a nasal decongestant with a water activity close to that of pure water, which comes as a 0.5 or 1 mg/ml Xylometazoline HCl solution in water, also comprising glycerol as humectant, benzalkonium chloride as preservative and a citrate buffer. BLOX4^®^ is a nose spray registered as medical device that relieves nasal allergic symptoms caused by pollen and house dust mite allergy (Andersson et al., 2008, 2011). It is a glyceryl monooleate based microemulsion with low water content, which forms a thin protective barrier on the nasal mucosa, claimed to provide immediate and long-lasting (several hours) effect. The long duration in situ is most probably due to the ability of monoglycerides, like glyceryl monooleate, to swell and form liquid crystalline phases when in contact with wet mucosa (Nielsen et al., 1998). Water sorption by the formulation will then inevitably also involve dehydration of the mucosa. As BLOX4^®^ is an oil-continuous microemulsion, we cannot determine its water activity. However, it behaves as a low water activity formulation when it swells. BLOX4^®^ was included in the study to investigate the potential use as a delivery vehicle for Xylometazoline HCl in comparison to Nasoferm^®^. Both formulations contain a range of excipients, which to some extent might influence the drug penetration over mucosa. However, the content of excipients in Nasoferm^®^ is most likely too low to have a major effect on water activity.

## Materials and methods

### Chemicals

^51^Chromium edetate (Chromium (51Cr) EDTA^®^, 3.7 MBq/ml solution for injection, GE Healthcare Ltd, UK) comprising 0.64 mg/ml ^51^Cr-EDTA was obtained from the department of Translational Medicine, Lund University/Skane University Hospital (SUS Malmö). Xylometazoline HCl (log D (pH 7.4) = 2.34, pKa = 10.6, *M*_W_ = 281 g/mol) (Golander & DeWitte, 1985), as well as polyethylene glycol 1500 (*M*_W_ = 1500 Da), sodium chloride (NaCl), disodium phosphate dihydrate (Na_2_HPO_4_·2H_2_O), monopotassium phosphate (KH_2_PO_4_), sodium hydroxide (NaOH), acetonitrile and methanol were all purchased from Sigma-Aldrich (Stockholm, Sweden). Disodium edetate (Na_2_-EDTA) (pKa = 2.0, 2.7, 6.2, 10.3, *M*_W_ = 372 g/mol) (Dawson, 1986) was obtained from Merck (Germany). 0.1 wt% Nasoferm^®^ (Nordic Drugs AB, Sweden) was obtained from a local Pharmacy and BLOX4^®^ (Bioglan AB, Sweden) was kindly provided by Bioglan AB. Ultra high quality (UHQ) water, purified at 25 °C by Elgastat UHQ II model UHQ-PS-MK3 (Elga Ltd., High Wycombe, Bucks, U.K.), was used in all in house preparations.

### Preparation of test formulations

Xylometazoline HCl (2.7, 4.5 and 8.1 wt%, respectively) was dissolved in phosphate-buffered saline (PBS, pH 7.4), prepared by mixing 130.9 mM NaCl, 5.1 mM Na_2_HPO_4_·2H_2_O, 1.5 mM KH_2_PO_4_ and adjusting pH with NaOH. Xylometazoline HCl (0.1-5 wt%) was also dissolved in BLOX4^®^ and compared to 0.1 wt% Nasoferm^®^ for reference. ^51^Cr-EDTA solutions were obtained by dissolving Chromium (51Cr) EDTA^®^ (3.2–32 µg/ml) and Na2-EDTA (0–200 mg/ml) in water. PEG1500 was added to decrease the water activity in the test formulations when applicable (65 wt% PEG1500 (aq) corresponds to a_w_ = 0.826 at *T* = 32 °C (Björklund et al., 2010)). All test formulations are listed in Tables 1 and 2.

**Table 1.** Xylometazoline HCl solubility versus degree of saturation (i.e. activity) in two donor formulations; 4.5 wt% Xylometazoline HCl in 65 wt% PEG (aq) gives the same saturation level as 8.1 wt% in PBS.

Vehicle	Water activity	Drug solubility wt%	Drug conc. wt%	Drug activity	Flux, J_ss_ (1-6h) µg/cm^2^h (J_ss_ ± 95% CI)
PBS	0.996*	11.9	2.7	0.22	1035 ± 159 (*n* = 24)
PBS			4.5	0.45	3091 ± 554 (*n* = 12)
PBS			8.1	0.68	3806 ± 451 (*n* = 6)
65 wt% PEG in PBS	0.826*	6.6	4.5	0.68	233 ± 97 (*n* = 7)
Nasoferm^®^	0.982	–	0.10	–	29.5 ± 9.0 (*n* = 6)^+^
BLOX4^®^	n.a.	–	0.10	–	2.4 ± 1.6 (*n* = 3)^+^
BLOX4^®^	n.a.	–	0.25	–	3.3 ± 0.5 (*n* = 3)
BLOX4^®^	n.a.	–	0.50	–	19.8 and 10.6 (*n* =2)
BLOX4^®^	n.a.	–	1.0	–	37.8 ± 2.5 (*n* = 3)
BLOX4^®^	n.a.	–	5.0	–	263.0 ± 21.7 (*n* = 2)

J_ss_: steady state flux.

*(Björklund et al., 2010)), ^+^Independent sample t-test showed a statistically significant difference between Nasoferm^®^ (*n* = 6, *M* = 29.5, *SD* = 12.3) and BLOX4^®^ flux (*n* = 3, *M* = 2.4, *SD* = 1.7), t (7) = 3.68, *p* = .008).

### Solubility measurements of drug formulations

The solubility of Xylometazoline HCl was determined by adding excess amount of drug to the formulations. The samples were then sealed and left stirring at 32 °C. After three days, they were filtered through a hydrophilic PTEE 0.45-µm filter and analyzed with HPLC-UV. Drug activity (a_D_) was calculated as the ratio between drug concentration in formulation and the drug solubility in the vehicle.

### Water activity measurements

The water activity of Nasoferm^®^ formulation in the present study was determined in triplicate with a bench-top water activity meter (LabTouch-aw, Novasina, Switzerland) and the mean value is provided in Table 1. The unit was calibrated with different solutions of standard saturated salts in the water activity range of interest in the present study.

### Preparation of porcine nasal mucosa membranes

Fresh porcine noses were obtained from Lund University as offal after surgical practice. Therefore, no additional ethical permit was required. The porcine noses were transferred to the lab immediately and stored in a freezer at −80 °C until use. Fresh samples were stored in refrigerator (<18 h) before tissue preparation. On preparation, the nose was split in two halves between the nostrils, separating the two nasal cavities. Left and right nasal cavity mucosa were handled equally. The mucosa was carefully separated from the underlying tissue using a scalpel and a tweezer. The nasal mucosa was then placed on a filter paper wetted with PBS-buffer and small membranes (Ø = 22 mm) were punched for the permeation study. Each membrane was code-marked with reference to its’ origin. The permeability experiments themselves were used to control the tissue viability and integrity. This has been reported to be a meaningful method to assess tissue viability as long as transport time was short before freeze storage of tissues (Shojaei, 1998; Nicolazzo et al., 2003). Potential deviation in flux between experiments was used as an indication, and data from a specific cell were disregarded based on two principles; either due to unreasonably high flux indicating damage to the membrane or for less pronounced variations outliers were excluded using Grubbs’ test.

### Flow-through cell diffusion studies

Diffusion experiments were conducted on flow through cells (PermeGear Inc. USA) (Bronaugh et al., 1986) at 32 °C, with excised nasal mucosa as membranes. The donor and receptor compartments of the diffusion cell are separated by a membrane (0.64 cm^2^). To avoid air bubbles the receptor media was degassed with nitrogen gas for 10 minutes before use. Before each experiment, the membranes were hydrated by placing them in the diffusion cells with PBS flowing in the receptor compartment for 1 h. Experiments were then initiated by adding formulation comprising Xylometazoline HCl (1 ml) and ^51^Cr-EDTA (128 µl), respectively. The donor cells were sealed with parafilm to avoid evaporation of water, the flow rate of receptor media (PBS, pH 7.4) was set to 1.5 ml/h and aliquots were collected every hour during a 6-hour period. Initially, samples were also collected after 30 and 90 minutes.

### Analytical methods

Xylometazoline HCl was analyzed at room temperature on a Varian 9012 (Agilent Technologies, USA) HPLC-UV (λ = 225 nm) instrument equipped with a Syncronis C8 column of dimensions 250 × 3 mm, 5 µm (Thermo Fisher Scientific, USA). Xylometazoline HCl concentrations were calculated from calibration curves of standard solutions in PBS (25-1000 µg/ml, R^2^ =1.00, LOD = 10 µg/ml). The retention time was 8 minutes using a mobile phase comprising acetonitrile (CH_3_CN) – water (35:65, v/v). The analysis was carried out at a wavelength of 225 nm and the flow rate of the mobile phase was 1 ml/min, and the injection volume was 20 µl. The radioactivity of ^51^Cr-EDTA was counted with an automatic gamma counter (1480 Wizard 3).

### Experimental considerations

A central part of this study is to study diffusion, while maintaining steady state conditions. This can be fulfilled when the gradients in water and model drug are kept constant by applying excess amounts of donor drug. When sink conditions are fulfilled, the driving force for diffusion will then be proportional to the concentration gradient. This is expressed in the generalized Fick’s first law of diffusion (Evans & Wennerström, 1999):

(1) $$J=-\;\frac{D\left(x\right)}{\mathrm{RT}}c\left(x\right)\frac{d\mu }{\mathrm{dx}}$$

where D(x)(cm^2^ h^−1^) is the diffusion coefficient at position x (cm), c(x) (µg cm^−3^) is the concentration of the diffusing molecule at position x, and dµ (J mol^−1^) is the chemical potential of the diffusing molecule. It is evident from eq. 1 that when steady state condition is reached, we would expect a linear dependence between flux and concentration (Aulton, 2007). Furthermore, drug release is diffusion-controlled (Fickian diffusion) when the fractional amount of drug released, up to 60%, is proportional to the square root of time (Ritger & Peppas, 1987). This is a useful representation for comparing drug delivery from different formulations.

Data from diffusion studies were analyzed from curves from cumulative permeated mass per membrane area as a function of time. The steady state flux, J_ss_ (µg/cm^2^h), could be calculated from the slope of the linear region of the curve. Data are presented as cumulative amount, as steady state flux of the model drug across the membrane, or as fraction of drug release (%) over time. Another important experimental aspect in this study is the effect of variations in water gradient on the diffusion coefficient. Thus, the gradient in drug chemical potential needs to be constant, while the gradient in water chemical potential is varied through addition of PEG1500 (Björklund et al., 2010). In order to achieve similar chemical potential of Xylometazoline HCl in formulations of PBS and 65% PEG1500 respectively, the concentration was chosen based on the same degree of saturation (drug activity (a_D_) = 0.68) concentration of Xylometazoline HCl in each formulation. At concentrations far below the saturation concentration, such as the case for Nasoferm^®^ and BLOX4^®^, the drug activity is assumed to be equal to the drug concentration (Atkins & De Paula, 2006; Aulton, 2007). The relation between chemical potential and activity can be realized from the following expression (Atkins & De Paula, 2006):

(2) $$\mu_{A}=\mu_{A}^{*}+\mathrm{RTln}\left(a_{A}\right)$$

where µ_A_ (J mol***^−^***^1^) is the chemical potential of A, µ*_A_ (J mol***^−^***^1^) is the chemical potential of pure A, R (J K***^−^***^1 ^mol***^−^***^1^) is the gas constant, T (°C) the temperature, and a_A_ the activity of A. Thus, in order to reach similar drug chemical potential in two different formulations, the drug activity needs to be considered at high concentration. While for very dilute concentrations, the actual concentration can be used for comparison (Atkins & De Paula, 2006).

### Statistical analysis

The data in the figures are given as mean values with error bars representing confidence interval (*p* = .05) for replicates at each time point. Statistical outliers were excluded based on two sided Grubbs’ test at *p* =.05. Statistical significance (*p* <.05) was tested using independent samples t-tests, and one-way analysis of variance (ANOVA).

## Results and discussion

### Verification of ex vivo method: Storage time and membrane origin

**Figure 1.**
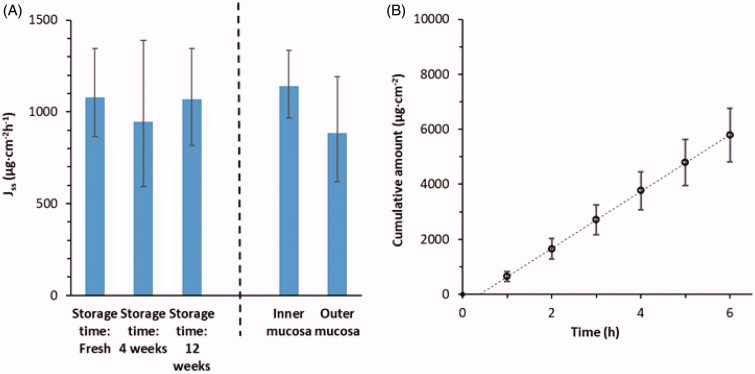
Effects on drug permeability through porcine nasal mucosa from local origin and storage at −80 °C were studied with donor formulations comprising 2.7 wt% Xylometazoline HCl in PBS. (A) Steady-state flux (1–6 h) across mucosa with respect to site of excision and storage time before use. (B) Cumulative amount of Xylometazoline HCl over time through randomly chosen mucosa membranes (*n* = 24). Bars indicate confidence interval, *p* = .05.

During the early development of the method, factors that could have an impact on the methodology were investigated with 2.7 wt% Xylometazoline HCl in PBS as test formulation. No major difference in drug permeability could be detected in mucosa retrieved from different pigs, used fresh (*n* = 7) or stored one month (*n* = 7) and three months (*n* = 10) at −80 °C before use (Figure 1(A)). This supports studies on buccal mucosa where it was shown that freezing of tissue did not affect drug permeability (Nicolazzo et al., 2003; Diaz del Consuelo et al., 2005). Neither could we detect any difference between mucosa from inner versus outer parts of the snout (Figure 1(A)). The steady-state flux of 2.7 wt% Xylometazoline HCl in PBS (pH = 7.4) over 1–6 hours where determined to 1035 ± 159 µg/cm^2^h (*n* = 24) (Figure 1(B), Table 1).

### Barrier response to ambient factors

**Figure 2.**
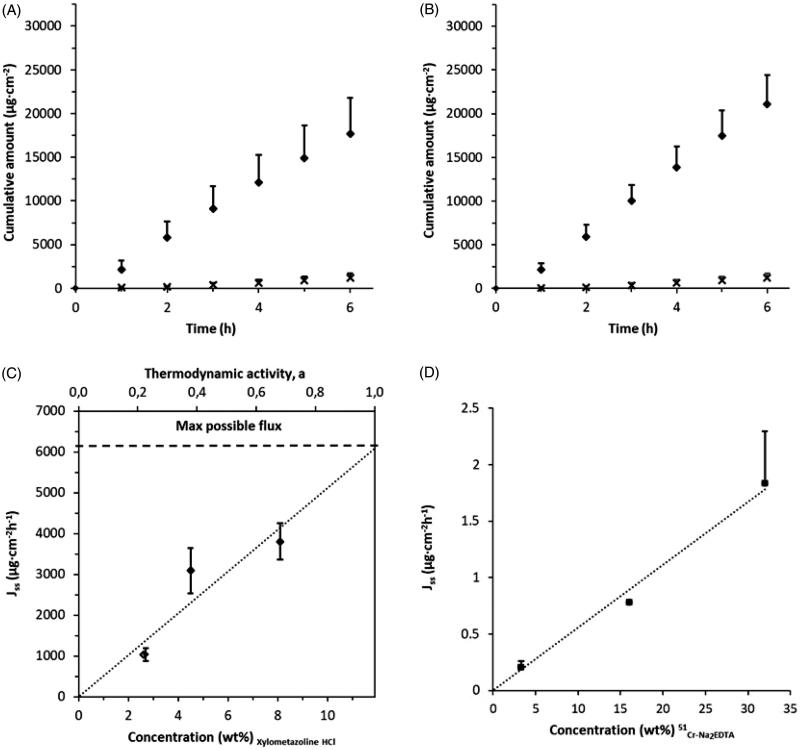
Effect of change in concentration and/or water activity gradient on drug permeability through porcine nasal mucosa. (A) Cumulative amount of Xylometazoline HCl over time obtained from two alternative vehicles, PBS (pH 7.4, *a*_w_ = 0.996, diamonds) and PBS mixed with 65% PEG (*a*_w_ = 0.826, crosses) (c.f. Table 1) comprising the same drug concentration, 4.5 wt% (*n* = 7 and 12 respectively). (B) Cumulative amount of Xylometazoline HCl over time obtained from the same donor formulations as in (A), comprising the same drug activity, *a*_D _= 0.68 (*n* = 6). (C) Effect of increasing the Xylometazoline HCl concentration on drug flux over mucosa with PBS as the vehicle (pH 7.4, *a*_w _= 0.996) (c.f. Table 1); the horizontal dashed line indicates the expected drug flux from a saturated solution, *a*_D_ =1 (*n* = 6-24). (D) Drug flux plotted against increased of ^51^Cr-EDTA concentration in water while maintaining a constant ^51^Cr-EDTA/Na_2_-EDTA ratio (µg/mg), (*n* = 2-4). The permeability is depending predominantly on ^51^Cr-EDTA concentration. Bars indicate confidence interval, *p* = .05.

Drug permeability over excised porcine nasal mucosa from Xylometazoline HCl (4.5 wt%) dissolved in two alternative vehicles with different water activity (Table 1) was investigated *ex vivo* in flow through diffusion cells. The results display a short lag time followed by a high and constant flux (Figure 2(A)). The steady-state flux, *J*_ss_ (1–6 h), obtained from the two vehicles differs by more than an order of magnitude (*J*_ss_ = 3091 vs 233 µg/cm^2^h; Table 1) where addition of PEG1500 (65 wt% a_w_ = 0.826 at *T* = 32 °C) appeared to be detrimental to drug transport. This is in line with our previous observations with oral mucosa, although the Xylometazoline HCl flux is about an order of magnitude higher through nasal mucosa from both vehicles (Ali et al., 2018).

However, the effect of thermodynamic activity on drug flux over a membrane also has to be taken into account when studying a particular drug in different formulations. The solubility of Xylometazoline HCl changes when adding PEG1500 to a PBS solution. Thus, maintaining equal drug concentration in the two different formulations will result in a difference in the driving force for diffusive transport (Björklund et al., 2010). Therefore, to confirm the hypothesis that the barrier properties of nasal mucosa respond to a change in ambient water activity, in a similar manner as skin and oral mucosa (Björklund et al., 2010; Ali et al., 2018), we adjusted the Xylometazoline HCl concentration to obtain the same drug activity (a_D_ = 0.68) in the two vehicles (i.e. 8.1 wt% in PBS and 4.5 wt% in PEG1500-PBS; Table 1). The results shown in Figure 2(B) confirm the difference in drug permability from the alternative formulations seen in Figure 2(A). The steady-state flux, *J*_ss_ (1–6 h), obtained from the two vehicles again differs by more than an order of magnitude (*J*_ss_ = 3806 vs 233 µg/cm^2^h; Table 1). By excluding the effect of different drug chemical potentials and absence of other excipients that may affect the permeability, it can be concluded that the negative effect on uptake of Xylometazoline HCl is due to the difference in water activity between the two formulations. The water activity of the solutions on either side of the tissues determines the water activity in the tissue (Björklund et al., 2010).

### *Ex vivo* model as predecessor for clinical trials

The steady-state flux over excised nasal mucosa of Xylometazoline HCl in PBS increases proportionally with concentration showing that absorption is driven by the drug activity in the formulation (Figure 2(C)). By extrapolating the trend line in Figure 2(C) to the saturation concentration of Xylometazoline HCl (11.9 wt%, a_w_ = 1, Table 1), the maximum drug flux from the current formulation can be estimated to 6091 µg/cm^2^h. Based on the findings presented in the previous section and the empirical fact that a water solution often proves to be the most effective vehicle for topical delivery ex vivo (Björklund et al., 2010), this flux can tentatively be taken as the target flux for developing any type of nasal formulation comprising Xylometazoline HCl. However, a water solution would most probably not survive long in the nose as it ought to be removed by the mucociliary clearance within about 15-20 minutes (Wadell, 2002; Davis & Illum, 2003).

Clinical trials are of course inevitable for developing effective nasal drug delivery systems, although the more background knowledge that can be gained ex vivo, and through explorative EVIVC studies the better. ^51^Cr-EDTA is identified as a suitable model drug to measure absorption across the epithelium (replacing in this case Xylometazoline HCl) as it has been used in several studies on physiology of the nasal epithelium before (Greiff et al., 1991, 1993, 1994; Andersson et al., 2011).

As a first step, we evaluated the concentration dependence of ^51^Cr-EDTA on permeability ex vivo. The steady state flux over excised nasal mucosa of ^51^Cr-EDTA in water increased proportionally with concentration (Figure 2(D)). The solution of ^51^Cr-EDTA was diluted from the original stock solution (Chromium (51Cr) EDTA^®^) by 20 times. It is thus fair to assume that ^51^Cr-EDTA is highly diluted and that at these concentrations the drug activity could be considered equal to the drug concentration and will be the main driving force for permeation.

**Figure 3.**
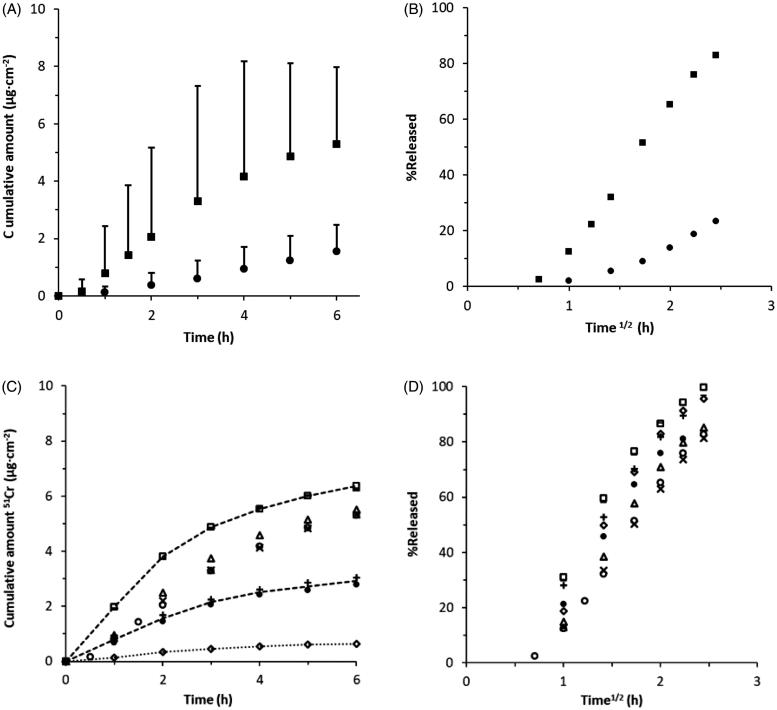
Effect of changing the water activity gradient or composition of formulation on ^51^Cr-EDTA permeability through porcine nasal mucosa using two alternative vehicles, water (*a*_w_ ∼ 1) and 65% PEG in water (*a*_w_ ∼ 0.826) (c.f. Table 1). (A) Cumulative amount of ^51^Cr-EDTA obtained over time from two alternative vehicles, water (*a*_w_ ∼ 1, squares) and 65% PEG in water (*a*_w_ ∼ 0.826, circles) comprising the same drug concentration, 32 µg/mg (*n* = 3) (c.f. Table 2). Bars indicate 95% confidence interval, *p* = .05. (B) Fraction of applied ^51^Cr-EDTA released versus the square root of time from the alternative vehicles in A. Almost all of the supplied ^51^Cr-EDTA penetrates from the water solution, while less than 20% is absorbed from the PEG-water mixture. (C) Cumulative amount of ^51^Cr-EDTA obtained over time with increasing Na_2_-EDTA concentrations from aqueous donor formulations. ^51^Cr-EDTA/Na_2_-EDTA- ratios (µg/mg) are shown as 3.2/10 (diamonds), 16/50 (plus signs and black filled circles), 32/0 (circles), 32/10 (triangles), 32/50 (bars), 32/100 (squares) and 32/200 (crosses), *n* = 2–4 (c.f. Table 2). Lines are appended as guidance for the eye. Significant differences (*p* < .05) between formulations with increasing ^51^Cr-EDTA concentrations were shown with one-way ANOVA (*p* < .05). (D) Fraction of applied ^51^Cr-EDTA released from aqueous donor formulation with increased Na_2_-EDTA concentrations versus the square root of time. The total data set shows that ^51^Cr-EDTA permeation scales with its concentration in the vehicle suggesting no effect of the added Na_2_-EDTA. There were no significant differences between the curves (*p* = 1.00) measured with one-way ANOVA (*p* < .05). N.B. The bar symbol (32/50) overlaps with the square symbol (32/100) in C and D.

Another important aspect to verify was of course if uptake of ^51^Cr-EDTA through mucosa follows the same water gradient dependence as Xylometazoline HCl. Figure 3(A) shows that decreasing the water activity of the formulation by addition of PEG1500 to the water solution while maintaining a fixed concentration of ^51^Cr-EDTA (32 µg/ml) do indeed result in a lower permeation. The steady state flux of ^51^Cr-EDTA was 1.26 µg/cm^2^h when administered in pure water and 0.18 µg/cm^2^h when supplied in the PEG1500-water mixture (Table 2). Figure 3(B) furthermore shows that while almost all of the supplied ^51^Cr-EDTA penetrates from the water solution, less than 20% is absorbed from the PEG1500-water mixture.

**Table 2.** Test formulations comprising ^51^Cr-EDTA are given below together with flux data (*J*_ss_) from Flow-through diffusion experiments.

Vehicle	Water activity	^51^Cr-EDTA conc. µg/ml	Na_2_-EDTA conc. mg/ml	Flux, *J*_ss_ (1-2h) µg/cm^2^h
65 wt% PEG in H_2_O	0.826^a^	32	0.1	0.18 ± 0.22 (*n* = 3)
H_2_O	1	32	0.1	1.26 ± 0.66 (*n* = 3)
		32	200.1	1.35 ± 0.50 (*n* = 3)
		32	100.1	1.83 ± 0.46 (*n* = 4)
		32	50.1	1.83 ± 0.23 (*n* = 3)
		16	50.05	0.78 ± 0.01 (*n* = 2)
		32	10.1	1.53 ± 0.28 (*n* = 4)
		3.2	10.01	0.21 ± 0.05 (*n* = 4)

a Not measured, assumed to be similar to water activity of 65 wt% PEG in PBS in Table 1.

The present results confirm that a change in the water gradient over nasal mucosa affect the permeability of both Xylometazoline HCl and ^51^Cr-EDTA in the same way. It means that ^51^Cr-EDTA ought to be a suitable radiolabeled molecule for studying uptake of hydrophilic molecules through mucosa from alternative vehicles *in vivo*. It furthermore suggests that flow-through diffusion cells can be used for evaluating the potential use of potent pharmaceuticals *ex vivo* and link these results to *in vivo* studies with healthy volunteers using a less harmful probe (^51^Cr-EDTA).

### Presence and potential use of Na_2_-EDTA

The product, Chromium (Cr-51) EDTA^®^, used as a source for ^51^Cr-EDTA in the present study comprised both radiolabeled ^51^Cr-EDTA and nonlabeled Na_2_-EDTA in equimolar amounts to secure that all ^51^Cr is complexed to EDTA. One obvious question is therefore if the presence of the closely related Na_2_-EDTA affects the permeation of ^51^Cr-EDTA through the mucosa. Another question that follows is if Na_2_-EDTA then can be used as absorption enhancer to adjust the permeation rate of ^51^Cr-EDTA and thereby allow a decrease in the amounts of radiolabeled compound required for conducting *in vivo* trials with healthy volunteers.

The influence of Na_2_-EDTA as a potential penetration enhancer for ^51^Cr-EDTA was investigated by increasing the amounts of Na_2_-EDTA while maintaining a constant ^51^Cr-EDTA to Na_2_-EDTA ratio, or by adding increasing amounts Na_2_-EDTA to a fixed concentration of ^51^Cr-EDTA in the aqueous donor solutions (Table 2). Figure 3(C) shows that drug flux increases with increasing concentrations at fixed ratios of ^51^Cr-EDTA (3.2-32 µg/ml) and Na_2_-EDTA (10–100 mg/ml). All formulations with equal ^51^Cr-EDTA concentration (32 µg/ml) created a cluster with similar permeation profiles and a steady-state flux up to 2 hours ranging between 1.26 and 1.83 µg/cm^2^h despite the fact that the concentration of Na_2_-EDTA was not the same (Figure 3(C) and Table 2). Figure 3(C) shows a trend of increase in drug flux increasing concentration. Significant differences (*p* < .05) were found between formulations with different ^51^Cr-EDTA concentrations. From the data shown in Figure 3(D), it is furthermore evident that all formulations have similar release kinetics which complies with 1^st^ order diffusion, and that most of the supplied ^51^Cr EDTA penetrates the mucosa during 6 h. Statistical analysis based on regression lines for cumulative amount released versus square root of time and one-way ANOVA analysis for significance (*p* < .05) using the slope and standard error of these curves showed that there were no significant differences between the curves (*p* = 1.00).

No major enhancing effect of Na_2_-EDTA could be detected for the penetration of ^51^Cr-EDTA, which indicates that the product, Chromium (Cr-51) EDTA^®^, is a feasible source for the radiolabeled probe required in our future proof of concept study and that the amount of Na_2_-EDTA present in the product does not interfere and can be ignored here.

### Products for administration of Xylometazoline HCl

**Figure 4.**
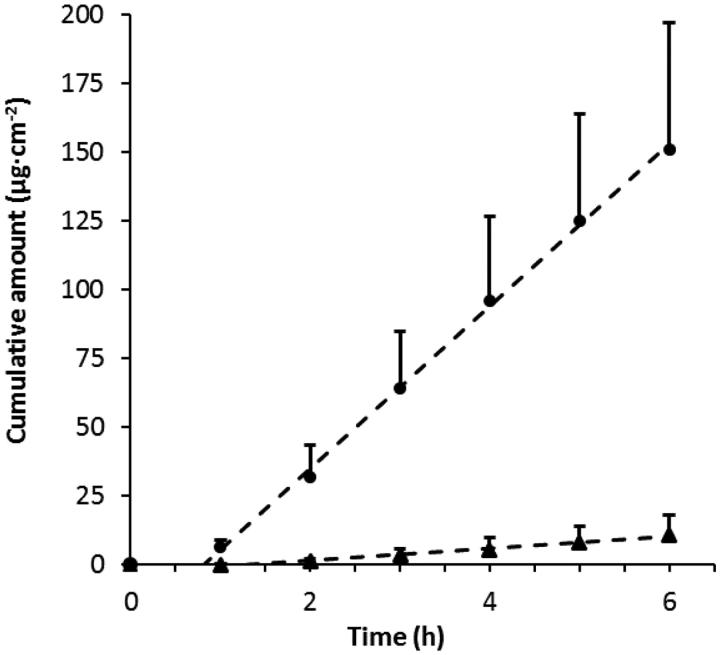
Xylometazoline HCl permeability through porcine nasal mucosa from two commercial vehicles, Nasoferm^®^ (circles, *n* = 6) and BLOX4^®^ (triangles, *n* = 3), both comprising 0.1 wt% drug (c.f. Table 1). An independent sample t-test showed a statistically significant difference between Nasoferm^®^ (*n* = 6, *M* = 29.5, SD = 12.3) and BLOX4^®^ flux (*n* = 3, *M* = 2.4, SD = 1.7), t(7) = 3.68, *p* = .008).

Drug diffusion through excised nasal mucosa for two formulations that are expected to differ considerably in how they affect the water gradient over nasal mucosa was investigated. Nasoferm^®^ 1 mg/ml (comprising an aqueous solution with high water activity, a_w_ = 0.982) and 1 mg/ml Xylometazoline HCl dissolved in BLOX4^®^ (comprising of a microemulsion that can absorb water from mucosa) were compared *ex vivo* (Figure 4). The steady-state flux differs by an order of magnitude where Nasoferm^®^ appears to be the better vehicle (*J*_ss_ = 29.5 and 2.4 µg/cm^2^h, respectively), and there was a statistically significant difference between Nasoferm^®^ (*n* = 6, *M* = 29.5, SD = 12.3) and BLOX4^®^ flux (*n* = 3, *M* = 2.4, SD = 1.7), t (7) = 3.68, *p* = .008). Noteworthy is however that both formulations are capable of dissolving higher amounts of drug. The microemulsion is capable of dissolving more than 50 mg Xylometazoline HCl per ml, which is reflected by the progressive increase in flux (e.g. 50 mg/ml ∈ 263 µg/cm^2^h, Table 1). A drug concentration of 0.1 wt% will then correspond to less than 2% of the maximum solubility. Nasoferm^®^ is not that different from the PBS-solution used above (maximum solubility = 11.9 wt%), with respect to overall composition (Table 1, Figure 2(A—B)). Hence, a drug concentration of 0.1 wt% would correspond to less than 1% of maximum solubility. As the drug is highly diluted in both formulation, the drug activity could be considered equal to the drug concentration (0.1 wt%), which will be the main driving force for diffusion. The observed difference in flux, can then be related to the gradient in water chemical potential.

The large difference between the two formulations is then the gradient in water activity over the mucosa, which they induce on application and the residence time in the nose. However, the duration time of Nasoferm^®^ is most probably hampered by fast mucociliary clearance. The high water sorption capacity of the microemulsion (BLOX4^®^) is expected to strongly favor duration in situ, while drug diffusivity over the mucosa may suffer due to dehydration. Whether good mucoadhesion or more hydrated mucosa is the factor that determines which formulation is the most efficacious has to be resolved in a clinical trial.

## Conclusions

In this work, we aimed to set up an *ex vivo* diffusion method to evaluate mucosal response to dehydration and resolve the hypothesis that mucoadhesion achieved through water sorption could also be a constraint for drug absorption via the nasal route. We further wanted to investigate whether this method could serve as a preclinical model to evaluate the potential use of potent pharmaceuticals ex vivo and link the results to in vivo studies on healthy volunteers using a less harmful probe (^51^Cr-EDTA).

We have shown that a modest increase in the water gradient over excised porcine nasal mucosa induces a substantial decrease in drug uptake for both Xylometazoline HCl and ^51^Cr-EDTA. The same result was obtained when comparing two vehicles on the market comprising Xylometazoline HCl; Nasoferm^®^ and BLOX4^®^.

Mucoadhesion based on water sorption can slow down drug uptake in the nasal cavity. A subsequent clinical study will determine whether prolonged duration of the vehicle *in situ* or preventing dehydration of the mucosa is the most important factor for improving bioavailability. The Chromium (51) EDTA^®^ is an acceptable substitute for Xylometazoline HCl in the foreseen study.
